# Effects of Boat Class and Size on Intracycle Velocity Variation During 2000 m Competitive Rowing: A GPS- and Accelerometry-Based Assessment

**DOI:** 10.3390/s26123745

**Published:** 2026-06-12

**Authors:** Joana Leão, Ricardo Cardoso, Aléxia Fernandes, Leandro Machado, Beatriz B. Gomes, Ricardo J. Fernandes

**Affiliations:** 1 Centre of Research, Education, Innovation and Intervention in Sport and Porto Biomechanics Laboratory, Faculty of Sport, University of Porto, 4200-450 Porto, Portugal; up202005623@edu.fade.up.pt (J.L.); davidrcardoso@gmail.com (R.C.); lmachado@fade.up.pt (L.M.); ricfer@fade.up.pt (R.J.F.); 2 CIPER, Faculty of Sport Sciences and Physical Education, University of Coimbra, FCDEFUC, 3040-248 Coimbra, Portugal; beatrizgomes@fcdef.uc.pt

**Keywords:** intracycle velocity variation, rowing, biomechanics, GPS, accelerometry, sensor-based monitoring, on-water assessment, technical efficiency

## Abstract

Rowing performance depends on boat velocity and technical efficiency, varying across boat classes. We quantified intracycle velocity variation (IVV) during 2000 m competitions using a GPS- and accelerometry-based monitoring and examined its relationship with biomechanical variables. Forty-nine races were recorded during three national regattas, involving 206 experienced rowers (72 females). Boats were classified by discipline (sweep vs. sculling) and length (short vs. long). Boat velocity and position were recorded using GPS (15 Hz) and accelerometry (100 Hz). Sculling boats demonstrated higher average velocity and lower IVV than sweep boats (*p* ≤ 0.05), possibly due to reduced lateral asymmetries. Long boats (quadruple scull, four and eight) reached significantly higher maximum, average, and minimum velocities than short boats (single scull, double scull, and pair) (all *p* ≤ 0.05), as well as greater technical index and distance per cycle. Correlation analysis identified large associations (r ≥ 0.5): in long boats, maximum and minimum velocity (r = 0.79) and cycle rate with distance per cycle (r = −0.50), whereas in short boats, average velocity was associated with minimum velocity (r = 0.76), technical index (r = 0.84) and distance per cycle (r = 0.64). In conclusion, IVV appears to be influenced by boat class and crew characteristics, representing a relevant sensor-derived indicator for monitoring technical efficiency in competitive rowing.

## 1. Introduction

Rowing is a cyclic sport in which boat velocity is the primary external indicator of performance [1,2,3]. Olympic rowing events are held over a 2000 m course and are divided into two disciplines: sculling (with two oars per rower) and sweep (with one oar per rower). In sculling, the Olympic classes comprise the single scull (one rower), double scull (two rowers) and quadruple scull (four rowers). In sweep rowing, the coxless pair (two rowers), coxless four (four rowers) and coxed eight (eight rowers plus coxswain) are included [4,5]. Across these different boat classes, technical efficiency plays a crucial role in determining boat velocity, with measurable differences observed depending on boat size and configuration [6,7,8,9].

Technical efficiency can be described as the ability to transfer propulsive work to the water while minimizing energy losses and resistive drag forces [10,11]. Biomechanical analysis may help enhance technical efficiency, increasing boat velocity [9,12,13]. However, research in rowing has mainly been conducted using ergometers and laboratory facilities, whereas on-water rowing presents substantial challenges for biomechanical data collection due to environmental constraints [14,15]. These challenges are further amplified in team boats, where the requirement to synchronize data across multiple rowers introduces additional technical and interpretive complexities [5,14,16]. In addition, feedback-oriented biomechanical monitoring has been proposed as a practical tool to address such complexities [17]. Therefore, sensor-based approaches that can capture boat motion under ecological and competitive conditions are particularly relevant for advancing applied rowing biomechanics.

Advances in biomechanical instrumentation and the development of new technologies, such as GPS and inertial sensors, have improved the accessibility, reliability and validity of data collection in contexts in which traditional laboratory methods are not feasible [3,14]. These systems allow continuous monitoring of boat displacement, velocity and acceleration during real on-water performance, providing information that cannot be fully reproduced in ergometer-based assessments. As a result, the quantitative analysis of technique in cyclic sports (such as swimming, canoeing and rowing) has gained increasing interest. [18,19,20,21]. The concept of the technical index, originally introduced in swimming, is defined as the product of velocity and distance per cycle. Higher technical index values generally reflect a greater capacity to maintain velocity while covering more distance per cycle and are therefore commonly used as an indirect indicator of technical performance in cyclic sports. A swimmer (or rower) with a high technical index covers more distance per cycle at higher velocity, which reflects superior propulsion [3,22]. Research on intracycle velocity variation (IVV) in swimming has demonstrated that lower IVV values are associated with more efficient movement patterns [23].

IVV has been investigated in relation to rowing performance, with larger variations in boat velocity within a cycle being associated with decreased movement efficiency [23,24,25]. From a sensor-based monitoring perspective, IVV is particularly relevant because it can be derived from continuous velocity and acceleration signals and may provide coaches with an objective measure of within-cycle boat stability. Biomechanical differences between rowing disciplines and boat classes may contribute to distinct patterns of boat velocity fluctuation. Sculling involves symmetrical force application through two oars, whereas sweep rowing relies on asymmetrical force production through a single oar, potentially influencing boat stability and within-cycle velocity changes. Similarly, boat size affects the mass and inertia of the system. Longer boats generally possess greater inertia, which may diminish velocity fluctuations throughout the cycle, while smaller boats are likely to be more sensitive to variations in force application and technical execution [26].

Although IVV has been associated with technical efficiency in rowing, its interaction with other biomechanical variables remains unknown, particularly under real competition conditions. Most previous studies have focused on laboratory assessments, training environments, individual boat classes, or isolated performance indicators. Consequently, limited information is available regarding how IVV and related biomechanical variables differ across Olympic rowing disciplines and boat sizes during official racing. Furthermore, the capacity of GPS- and accelerometry-derived variables to discriminate biomechanical behavior between boat classes has received little attention. Addressing these gaps may improve the understanding of technical efficiency in rowing and provide coaches with practical tools for performance monitoring. Therefore, the present study examined IVV and other biomechanical variables across Olympic rowing boat classes during official 2000 m competitions.

The aim of the current study was to assess IVV and other relevant biomechanical variables in Olympic boat classes during official 2000 m competitions using GPS and accelerometry and to examine how they differ across rowing disciplines and boat sizes. We hypothesized that IVV would significantly differ between rowing disciplines and boat sizes, with sculling and long boats showing lower IVV and more efficient biomechanical patterns than sweep and short boats.

## 2. Materials and Methods

A total of 49 races were recorded during three national regattas held at a high-performance rowing venue, involving 206 experienced rowers (72 females) with considerable experience in national and international regattas, and regular training volumes of 10–20 h per week. The unit of analysis was the race. No boats were represented more than once in the dataset, ensuring independence among observations. The sample included 12 male and 10 female single sculls, 10 male and 7 female double sculls, 6 male and 5 female quadruple sculls, 6 male and 2 female pairs, 6 male fours and 6 female fours, 6 male and 4 female eights. For analysis purposes, the pair, four and eight were grouped as sweep rowing boats, while the single sculls, double sculls and quadruple sculls were grouped as sculling boats. In addition, the quadruple scull, four and eight were classified as long boats, whereas the single sculls, the double sculls and pairs were considered short boats.

Boat position, velocity and acceleration were obtained using a 15 Hz GPS unit with 12 channel receivers integrated with a tri-axial accelerometer sampling at 100 Hz (GPSPORT, Canberra, Australia), mounted on the bow deck of each boat [3,27,28,29]. This configuration enabled simultaneous recording of boat displacement from GPS data and high-frequency longitudinal acceleration from the accelerometer, allowing the extraction of race- and cycle-related biomechanical variables under real competition conditions. The GPS provided displacement data for calculating boat velocity, while the accelerometer captured longitudinal acceleration patterns, allowing the assessment of rowing cycle rate and IVV. Sensor placement was standardized for all measurements and devices were calibrated according to the manufacturer’s instructions before data collection. The bow deck was selected to minimize interference with rowing execution and to provide a consistent reference point for boat motion across all classes. Environmental conditions at the start and finish of each race, including wind velocity, direction, air temperature and relative humidity, were recorded using a portable weather station (NK 5400, Kestrel, Boothwyn, PA, USA) positioned at water level. Races were held with wind velocities between 5 and 10 km·h^−1^. Preliminary analyses indicated no systematic differences in these variables between boat classes or sexes; therefore, they were not included as covariates in the final analyses. All participants provided written informed consent in accordance with the local research ethics committee and the Declaration of Helsinki.

Data were analyzed using a validated custom MATLAB algorithm (version 2023a; MathWorks Inc., Natick, MA, USA) [27] adapted for rowing analysis, allowing to assess maximum velocity (highest velocity registered during the race), minimum velocity (lowest velocity registered after the maximum velocity is achieved), average velocity (ratio of distance covered to the time taken), time to peak (time since the beginning of the race until maximum velocity is achieved), technical index (product of distance per cycle and velocity), cycle rate (mean number of rowing cycles per minute), distance per cycle (ratio of mean velocity to the corresponding stroke rate), IVV (difference between maximum and minimum velocity in each cycle) and relative IVV (ratio between IVV and average velocity). Rowing cycles were identified from the longitudinal acceleration signal, and GPS-derived velocity was used to quantify velocity-related outcomes throughout the race. The combination of both signals allowed IVV to be calculated as a cycle-by-cycle indicator of boat velocity fluctuation, while preserving the ecological validity of official competition data.

All variables were calculated as mean ± SD values for the complete race and per 100 m splits. Data normality and homogeneity were verified using the Kolmogorov–Smirnov and Levene tests, respectively. All statistical procedures were performed using SPSS 27 (Chicago, IL, USA) with the significance level set at *p* ≤ 0.05. A two-way analysis of variance (ANOVA) was used to examine the main effects of boat discipline (sweep vs. sculling) and sex (male vs. female), as well as the interaction effect between these factors, on the calculated variables. When significant main effects were identified, post hoc pairwise comparisons with Bonferroni adjustment were applied. For each ANOVA, effect sizes were calculated using partial eta squared (η^2^) and interpreted as small (η^2^ = 0.01), medium (η^2^ = 0.06) or large (η^2^ ≥ 0.14). The Pearson correlation coefficients were used to evaluate linear relations between variables and interpreted as large (0.5–1.0), medium (0.3–0.5) and small (0.1–0.3). Heatmaps of velocity, cycle rate and IVV were generated over a 2000 m distance using MATLAB. Data were averaged in 100 m segments and color-coded to enable visual comparison of performance patterns derived from the sensor recordings across the race distance [30].

## 3. Results

Mean ± SD values of the selected biomechanical variables across rowing discipline (sweep vs. sculling) and boat size (long vs. short) for male and female crews during the 2000 m competition are presented in Table 1. Sculling boats achieved higher average velocity and lower IVV compared with sweep boats, with no differences being observed for maximum velocity, minimum velocity, technical index, cycle rate, time to peak velocity or distance per cycle. Male crews presented greater maximum, average and minimum velocities, higher technical index, longer distance per cycle and greater IVV than female crews, with no significant interaction effects between rowing discipline and sex. Long boats presented higher maximum, average and minimum velocities, technical index, distance per cycle and lower IVV compared with short boats. No significant interaction effects between boat size and sex were detected, except for cycle rate. These findings indicate that GPS- and accelerometry-derived variables were able to discriminate biomechanical behavior according to both rowing discipline and boat size during official 2000 m racing.

**Figure 1:**
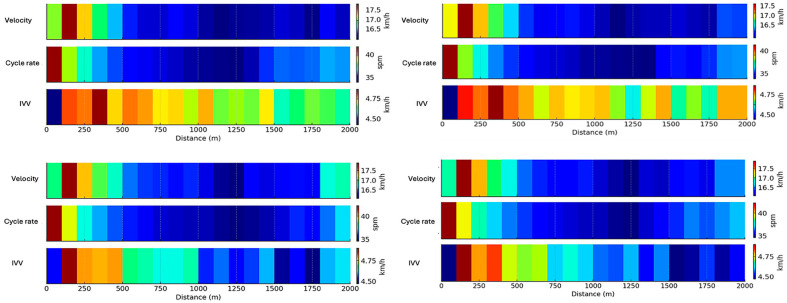
Distribution of velocity, cycle rate and intracycle velocity variation during a 2000 m competition for different boat classes: sculling, short, sweep and long boats (**upper**-**left**, **upper**-**right**, **lower**-**left** and **lower**-**right** panels, respectively) and each colored rectangle represents a 100 m segment. The heatmaps were generated from GPS- and accelerometry-derived variables averaged over successive race segments.

Heatmaps representing the distribution of velocity, cycle rate and IVV over the 2000 m race are presented in Figure 1. The relationships between biomechanical variables according to boat discipline and boat size are displayed in Table 2. In sculling, maximum velocity showed large associations with average velocity, minimum velocity, technical index and distance per cycle. Average velocity also showed large associations with minimum velocity and distance per cycle. In sweep boats, maximum velocity presented moderate associations with average velocity, minimum velocity and technical index, while the relationship between average and minimum velocity was also moderate. In long boats, maximum velocity showed large associations with minimum velocity, technical index and distance per cycle and a moderate relationship with average velocity. Average velocity also showed large associations with minimum velocity and distance per cycle. In short boats, maximum velocity demonstrated large associations with average velocity, minimum velocity, technical index and distance per cycle, with average velocity also showing large associations with minimum velocity and distance per cycle. Overall, the correlation patterns suggest that sensor-derived velocity and cycle-related variables provide complementary information on performance and technical efficiency across boat configurations.

## 4. Discussion

The current results highlight the relevance of boat discipline and size for characterizing biomechanical variables and performance outcomes during competitive rowing. Using a GPS- and accelerometry-based monitoring approach, this study showed that boat motion variables obtained under official racing conditions can provide meaningful information on technical efficiency across Olympic rowing classes. Data revealed that sculling boats demonstrated higher average velocity and lower IVV than sweep boats. In rowing, increased water resistance is associated with higher IVV values, whereas maintaining a more constant velocity may enhance efficiency [3,23]. This suggests that sculling promotes a more stable propulsion, which can be possibly attributed to the symmetrical contribution of two oars per rower and reduced lateral asymmetries [7,25,31]. Relative IVV reflects the extent to which fluctuations in velocity influence average velocity. Lower relative IVV was observed in sculling boats, which possibly means they are achieving that velocity with less wasted energy compared to sweep boats [3,19]. From a monitoring perspective, this supports the use of IVV as a sensitive variable for identifying differences in boat stability and propulsion continuity between rowing disciplines.

When comparing boat disciplines across sexes, male crews exhibited higher values in maximum, average, and minimum velocities, as well as in technical index, distance per cycle, and IVV. These differences are consistent with well-documented sex-related disparities in physiological and biomechanical determinants of performance, including greater absolute muscle strength, higher power output, superior maximal oxygen uptake, and more favorable anthropometric characteristics in male rowers [31,32,33]. In rowing specifically, these factors contribute to an increased capacity to generate and sustain propulsive force throughout the rowing cycle, thereby enhancing both boat velocity and efficiency-related indicators. Despite these differences, no significant interaction effects were found between boat discipline and sex. This suggests that while absolute performance differs, the differences observed between sculling and sweep rowing are consistent across sexes. Therefore, the sensor-derived variables used in the current study appear to capture boat discipline effects that are not dependent on sex-specific performance level.

Data revealed that long boats reached higher maximum, average and minimum velocities, which could be mainly attributed to the greater number of rowers contributing to propulsion. Long boats demonstrated a higher technical index, cycle rate and distance per cycle, as well as lower IVV, which may be related to both biomechanical and hydrodynamic advantages. Longer hulls might allow more efficient transfer of mechanical power into boat propulsion, reducing wave drag and minimizing IVV and relative IVV [6,26,34,35]. The combined effect of increased propulsive force from multiple rowers and the stabilizing influence of a longer hull enables sustaining higher velocity throughout the race [31]. The lower IVV observed in long boats also indicates that GPS- and accelerometry-derived measures can reflect the stabilizing effect of boat configuration on within-cycle velocity fluctuations.

When comparing male and female crews across different boat sizes, differences were observed in all variables except for cycle rate. Female crews might compensate for lower absolute power by increasing cycle rate. However, this approach may not be the most efficient, since previous research has emphasized the importance of adjusting cycle rate according to power output to maintain technical efficiency and optimize overall performance [3,15,36,37]. In addition, no significant interaction effects were found between boat size and sex, indicating that the advantages of long boats apply similarly to both male and female crews. This suggests that while absolute power and strength differ between sexes, the biomechanical and hydrodynamic benefits of longer hulls benefit all crews equally. This reinforces the applicability of sensor-based boat motion analysis across male and female crews when the aim is to compare technical behavior rather than absolute performance alone.

The analysis of the 100 m race segments throughout the competition revealed that biomechanical variables behaved differently over the 2000 m. It was observed that sculling and long boats maintained higher average velocity and more stable IVV than sweep and short boats, respectively. Velocity showed an initial increment followed by a progressive decline across all boat classes, with sculling and long boats maintaining higher values than sweep and short boats, respectively. Sweep oars have a larger surface area since each rower uses only one oar. As a result, the bigger blade generates greater impulse, which, over the course of a race, may compromise the ability to maintain a steady boat velocity [38]. Cycle rate decreased over the race in all conditions, possibly due to fatigue accumulation [30,39]. IVV maintained more stable values for long boats throughout the distance, possibly due to the stabilizing effect of longer hulls [23,24,25,34]. The segmental heatmap analysis adds practical value by showing how sensor-derived variables evolve across the full race distance, rather than only providing a single mean value for the 2000 m effort.

Data evidenced that some large associations were specific to each rowing discipline. In sculling, large associations were observed between maximum velocity, average velocity and distance per cycle, as well as between average velocity and technical index, distance per cycle and IVV. These associations may be explained by the symmetrical use of two oars in sculling, which ensures balanced force application and minimizes lateral asymmetries. This symmetry produces a more uniform propulsion pattern, contributing to steadier boat velocity and supporting the observed relationships with the technical index and distance per cycle [40]. However, some large associations, such as those between maximum velocity and minimum velocity, technical index and IVV, as well as between minimum velocity, technical index and distance per cycle, were present in both disciplines. This reflects that some biomechanical relationships are fundamental to rowing performance and independent of boat discipline [17,41]. Accordingly, the combined interpretation of velocity, cycle rate, distance per cycle and IVV may provide a more complete sensor-based profile of rowing performance than isolated variables.

Long boats presented a large relation between maximum velocity and minimum velocity and a large inverse relation between cycle rate and distance per cycle. This relation may be explained by the greater inertia and hydrodynamic drag resulting from the longer hull and the higher number of rowers, which together increase the overall mass and resistance of long boats [31,42]. In short boats, average velocity had a large association with minimum velocity, technical index, and distance per cycle. The lower number of rowers may explain why average velocity had a large association with technical index, since even small differences in power application or disparities in cycle coordination between rowers are more rapidly reflected in velocity [1,43,44]. Moreover, in short boats, longer cycles are likely related largely to average velocity, likely due to the lighter hull and reduced hydrodynamic drag, enabling each cycle to generate immediate acceleration and sustain velocity [44]. These findings suggest that sensor-based monitoring may be particularly useful in short boats, where small technical variations can have a more immediate effect on boat velocity.

From a practical perspective, these findings suggest that sensor-derived IVV monitoring can provide coaches with a sensitive indicator of technical efficiency during competition. Therefore, coaches may use IVV analysis to identify technical inefficiencies, support crew selection and optimize cycle rate strategies according to boat class and race segment. Because the variables analyzed in the current study were obtained from boat-mounted GPS and accelerometry, this approach can be applied without interfering with rowing execution and may be suitable for repeated monitoring during training and competition. Integrating IVV monitoring with other biomechanical variables may support individualized feedback and contribute to more effective training interventions across Olympic boat classes. In applied settings, the combined analysis of average velocity, minimum velocity, cycle rate, distance per cycle and IVV may help coaches identify whether performance differences are mainly related to propulsion magnitude, cycle organization or within-cycle velocity stability.

However, a limitation of the current study is the absence of data from the women’s four, a boat class not represented in the regattas analyzed. This gap restricts the generalization of the findings to all Olympic categories and should be addressed in future research. A further limitation is that the present approach focused on boat motion and did not include direct measurements of oarlock force, rower power output or individual rower coordination. Therefore, although GPS and accelerometry provided relevant information on boat behavior, they do not fully explain the mechanical sources of the observed velocity fluctuations. In addition, future studies could integrate power output measurements to better understand the relationship between IVV, technical efficiency and performance, ultimately contributing to the development of normative values for each crew and boat class. Future research should also examine the reliability and sensitivity of sensor-derived IVV across repeated trials, environmental conditions and training phases, particularly if this variable is to be used for longitudinal monitoring. Furthermore, larger-scale and longitudinal investigations are warranted to develop normative data and evidence-based reference ranges, thereby strengthening the practical utility of IVV for athlete monitoring, performance evaluation, and informed coaching decision-making.

## 5. Conclusions

The current study demonstrated that boat discipline and size substantially influence biomechanical variables and performance outcomes during competitive rowing. Using GPS- and accelerometry-derived measures collected during official 2000 m races, the current findings showed that boat-mounted sensors can provide relevant information on velocity behavior, cycle characteristics and IVV across Olympic boat classes. Sculling and long boats were associated with higher velocities, greater technical efficiency indicators and lower IVV than sweep and short boats. Male crews achieved superior absolute values across most variables, although no significant interaction effects with boat discipline or size were observed, indicating that the biomechanical and hydrodynamic benefits of sculling and long boats apply consistently across sexes. These findings highlight IVV as a relevant performance-related indicator and reinforce the importance of considering both boat characteristics and crew composition when analyzing rowing performance. Overall, sensor-derived IVV appears to be a useful marker for monitoring technical efficiency and within-cycle boat velocity stability in real competitive rowing conditions.

## Figures and Tables

**Table 1:** Biomechanical variables according to boat discipline (sweep vs. sculling) and size (long vs. short) during the 2000 m competition in male and female crews.

	Sweep		Sculling		Long		Short	
Variable	Male	Female	Male	Female	Male	Female	Male	Female
Maximum velocity (km/h)	22.09 ± 1.64 ^c, α^	19.61 ± 1.56 ^α^	22.65 ± 1.66 ^c, α^	18.86 ± 2.40 ^α^	22.89 ± 1.53 ^c, α^	20.56 ± 1.45 ^α^	21.94 ± 1.70 ^b, c, α^	18.85 ± 1.66 ^b, α^
Average velocity (km/h)	16.34 ± 1.13 ^c, α^	14.09 ± 1.46 ^α^	17.13 ± 1.49 ^a, c, α^	15.94 ± 0.67 ^a, α^	17.33 ± 1.53 ^c, α^	15.95 ± 3.09 ^α^	16.22 ± 1.01 ^b, c, α^	13.8 ± 0.94 ^b, α^
Minimum velocity (km/h)	12.3 ± 1.75 ^c, α^	10.16 ± 1.46 ^α^	12.39 ± 1.34 ^c, α^	10.91 ± 0.69 ^α^	12.85 ± 1.05 ^c, α^	11.2 ± 0.64 ^α^	12.01 ± 1.79 ^b, c, α^	9.9 ± 1.41 ^b, α^
Time to peak (s)	29.88 ± 74.66 ^α^	13.36 ± 7.81 ^α^	26.66 ± 44.88 ^α^	15.08 ± 5.36 ^α^	27.15 ± 44.78 ^α^	12.93 ± 2.92 ^α^	29.58 ± 74.71 ^α^	14.1 ± 7.81 ^α^
Technical index (m^2^/s cycle)	36.68 ± 4.05 ^c, α^	28.77 ± 3.99 ^α^	37.85 ± 4.73 ^c, α^	28.29 ± 3.89 ^α^	39.11 ± 4.02 ^c, α^	30.98 ± 2.58 ^α^	35.39 ± 3.84 ^b, c, α^	27.58 ± 4.00 ^b, α^
Cycle rate (cycles.min^−1^)	34.86 ± 2.18 ^c, β^	33.69 ± 3.47 ^α^	36.85 ± 2.97 ^c, β^	35.09 ± 4.34 ^α^	35.85 ± 2.33 ^α^	35.18 ± 3.38 ^α^	35.48 ± 3.18 ^b, c, α^	32.95 ± 3.08 ^b, α^
Distance per cycle (m)	7.84 ± 0.37 ^c, α^	7.1 ± 0.71 ^α^	7.70 ± 0.61 ^c, α^	7.21 ± 0.48 ^α^	7.97 ± 0.97 ^c, α^	7.33 ± 0.33 ^α^	7.68 ± 0.37 ^b, c, α^	7.03 ± 0.77 ^b, α^
IVV (km/h)	5.38 ± 0.93 ^c, α^	4.67 ± 1.60 ^α^	4.92 ± 0.98 ^a, c, α^	3.83 ± 0.82 ^a, α^	4.87 ± 0.88 ^c, α^	4.95 ± 1.62 ^α^	5.14 ± 0.98 ^b, c, α^	4.07 ± 1.16 ^b, α^
Relative IVV (%)	28.89 ± 6.26	24.50 ± 4.59	33.36 ± 5.55 ^a, c, α^	36.00 ± 9.98	28.28 ± 5.56	25.67 ± 6.56	33.75 ± 5.76 ^b, c, α^	38.17 ± 9.00

^a^, ^b^ and ^c^: differences from sweep, long and females (respectively) for *p* ≤ 0.05. Eta square: ^α^: [0.25–0.50]; ^β^: [0.51–0.75]; #: [0.76–1].

**Table 2:** Associations between biomechanical variables in sweep and sculling boat disciplines during a 2000 m competition.

Variables	Average Velocity (km/h)		Minimum Velocity (km/h)		Time to Peak (s)		Technical Index (m^2^/s·Cycle)		Cycle Rate (Cycles.min^−1^)		Distance Per Cycle (m)		IVV (km/h)		Relative IVV (%)	
	A	B	A	B	A	B	A	B	A	B	A	B	A	B	A	B
Maximum velocity (km/h)	0.49 */ 0.37	0.79 */ 0.79 *	0.66 */ 0.79 *	0.58 */ 0.48	0.14/ 0.19	0.13/ 0.12	0.69 */ 0.65 *	0.80 */ 0.77 *	0.59 */ 0.49 *	0.33 */ 0.37 *	0.32/ 0.15	0.55 */ 0.65 *	0.36/ 0.42 *	0.22/ 0.29 *	0.18/ 0.21	0.16/ −0.04
Average velocity (km/h)			0.37 /0.47 *	0.84 */ 0.76 *	0.02 /0.05	0.19/ 0.20	0.47 */ 0.38 *	0.86 */ 0.90 *	0.21/ 0.12	0.42 */ 0.54 *	0.24/ 0.16	0.68 */ 0.55 *	0.33/ 0.37	0.11/ 0.13	0.21/−0.165	0.57/ −0.34 *
Minimum velocity (km/h)					0.00/ 0.00	0.17/ 0.16	0.58 */ 0.76	0.71 */ 0.61	0.21/ 0.38	0.42 */ 0.44 *	0.24/ 0.39	0.55 */ 0.46 *	0.33/ 0.31	0.03/ 0.02	−0.21/ 0.03	0.27/ −0.33 *
Time to peak (s)							0.27/ 0.20	0.13/ 0.15	−0.18/ −0.13	0.14/ 0.12	0.37/ 0.34	0.09/ 0.11	0.32/ −0.23	−0.02/ −0.04	0.125	−0.04/ −0.13
Technical index (m^2^/s· cycle)									0.03/ −0.10	0.14/ 0.16	0.72 */ 0.66 *	0.84 */ 0.75 *	0.07 /0.19	0.11/ 0.17	−0.18/−0.03	0.03/ −0.257
Cycle rate (cycles.min^−1^)											0.09/ −0.50 *	−0.27/ −0.2	0.26/ 0.01	−0.1/0	0.25/ −0.08	−0.12/ −0.19
Distance per cycle (m)													−0.04/ 0.16	0.08/ 0.09	0.24/ 0.06	0.05/ −0.17
IVV (km/h)															0.88 */ 0.86 *	0.926 */ 0.87 *

* Significant correlation for *p* < 0.05. A and B represent sweep long and short sculling boats, respectively.

## Data Availability

The data presented in this study are available within the article.
